# Care Aides Compassion Fatigue, Burnout, and Compassion Satisfaction Related to Long-Term Care (LTC) Working Environment

**DOI:** 10.1177/07334648251328400

**Published:** 2025-03-24

**Authors:** Ashikur Rahman, Yinfei Duan, Holly Symonds-Brown, Jordana Salma, Carole A Estabrooks

**Affiliations:** 1 Faculty of Nursing, 3158 University of Alberta, Edmonton, AB, Canada

**Keywords:** compassion fatigue, compassion satisfaction, burnout, long-term care, COVID-19, care aides

## Abstract

Severe staff shortages, sustained stress, low compassion satisfaction, high compassion fatigue, and serious levels of burnout among healthcare workers were frequently reported during COVID-19. In this cross-sectional study with 760 care aides working in 28 LTC homes in Alberta, Canada, we used a two-level multilevel regression model to examine how working environments were associated with compassion fatigue, burnout, and compassion satisfaction measured with the Professional Quality of Life (ProQOL-9) scale. Our findings showed that higher compassion satisfaction and lower burnout were observed when care aides perceived a more supportive working culture. Care aides reported higher compassion fatigue when there was a lack of structural or staffing resources. We also found that perceptions of not having enough staff or enough time to complete tasks were significantly associated with higher levels of burnout. These findings suggest which elements of the working environment may be promising targets for improvement efforts.

**What this paper adds**

• Provides a richer understanding of compassion fatigue among care aides in long-term care (LTC) settings.
• Addresses a research gap on LTC working environments, focusing on which elements of the working environment influence care aides’ compassion fatigue, burnout, and compassion satisfaction.
• Offers contextualized insight into how individual, unit, facility, and organizational factors can be predictable for burnout, compassion fatigue, and compassion satisfaction in LTC settings.

**Application of study findings**

• This research finding highlights the importance of fostering workforce resilience through a supportive work culture, sufficient staffing, and structural resources.
• Supportive work culture emerges as a modifiable environmental factor that is both economically viable and readily implementable in LTC settings. This environmental modification can mitigate the threats of workplace burnout and improve compassion satisfaction among the care aides.
• The findings could inform policy changes at both organizational and governmental levels to improve working conditions in LTC homes through regular assessments of workplace culture and resources.

## Background

The psychological well-being of healthcare professionals has increasingly become a significant concern over the past 15 years (Bamforth et al., 2023). This well-being is profoundly influenced by compassion-related work life, a term encompassing physical, mental, and emotional challenges healthcare workers encounter in their daily interactions with patients. This concept includes both positive aspects, such as compassion satisfaction, and negative aspects, like compassion fatigue and burnout (Stamm, 1995, 2010). Compassion satisfaction is the pleasure that derives from effectively helping others or motivating them to offer help (Meadors et al., 2010). It inspires healthcare workers to improve patient care and believe they can make a positive impact (Slatten et al., 2011). Compassion fatigue, on the other hand, results from the stress of working in challenging situations or witnessing others’ pain or distress (Figley, 2013; Sorenson et al., 2016; Yu et al., 2016). For instance, oncology nurses often experience compassion fatigue after repeated exposure to patients’ suffering from the side effects of aggressive chemotherapy or those in the end stages of cancer (Potter et al., 2010). It is also called the “cost of caring” in the sense of negative consequences of caring for patients (Figley, 2002; Figley & Roop, 2006).

Burnout, an important element of compassion-related work life, significantly impacts healthcare professionals’ ability to provide empathetic and effective care. It is characterized by prolonged feelings of emotional exhaustion, hopelessness, and difficulties in performing jobs effectively (Braunschneider, 2013; Bride et al., 2007; Cañadas-De la Fuente et al., 2018; Slatten et al., 2011; Stamm, 1995). Burnout and compassion fatigue are interrelated; however, the development of burnout is gradual, while compassion fatigue can occur more rapidly (El-Bar et al., 2013; Hunsaker et al., 2015; Todaro-Franceschi, 2019). During COVID-19, many studies frequently reported severe staff shortages, sustained stress, low compassion satisfaction, high compassion fatigue, and serious levels of burnout among healthcare workers (Galanis et al., 2021; Garnett et al., 2023; Lluch et al., 2022; Richert & Zucchero, 2023; Sexton et al., 2022). A recent study in the United States (US) revealed that nearly half of the US healthcare workers experienced burnout and were searching for new jobs (Rotenstein et al., 2023).

In Canada, unregulated care aides or personal support workers constitute approximately 90% of the long-term care (LTC) workforce (Chamberlain et al., 2019). These workers are responsible for managing physical symptoms and providing emotional support to residents with complex needs (Fujisawa & Colombo, 2009; White et al., 2021). The COVID-19 pandemic exacerbated already difficult working conditions, introducing severe staffing shortages, severe resident isolation, and increased resident mortality (Titley et al., 2023; Wagner, 2020). Care aides experienced physical and emotional exhaustion due to heavy workloads (Reinhardt et al., 2022), staffing crises (Blanco-Donoso et al., 2021; Franzosa et al., 2022), inadequate infrastructure (Estabrooks et al., 2020), and insufficient safety measures (Estabrooks et al., 2020). Healthcare workers caring for COVID-19 patients reported higher levels of burnout, compassion fatigue (Cheung et al., 2022; Ruiz-Fernández et al., 2020), and secondary trauma (Egol et al., 2011; Vagni et al., 2020). Studies have shown that LTC nurses experience higher compassion fatigue when unable to provide sufficient support to residents (Kolthoff & Hickman, 2017; Shahar et al., 2019). However, research indicates that a supportive working environment can mitigate these negative outcomes, reducing compassion fatigue and burnout (Wu et al., 2016) and turnover intention (Marshman et al., 2022). Conversely, staffing shortages can increase workload, leading to higher burnout and lower job satisfaction (Kelly & Todd, 2017; Lee et al., 2020).

Identifying the elements of safe and supportive LTC working environments is essential if we are to make progress on reducing the negative consequences of compassion-related work life. The aim of this study is to investigate which elements of the LTC working environment are related to care aides compassion fatigue, burnout, and compassion satisfaction. To date, most empirical studies have investigated prevalence and levels of compassion fatigue (Hunsaker et al., 2015; Kartsonaki et al., 2023; Van Mol et al., 2015; Xie, Chen, et al., 2021; Xie, Wang, et al., 2021) and contributing factors (Oktay & Ozturk, 2022; Sprang et al., 2007; Xie, Wang, et al., 2021) among physicians and nurses in acute healthcare settings, for example, emergency, oncology, and intensive care units. Although substantial research addresses burnout in LTC settings (Chamberlain et al., 2017; Cooper et al., 2016; Duan et al., 2023; Gruneir et al., 2024), we located little evidence exploring compassion fatigue and its relationship with the working environment, revealing a critical gap in the literature. A better understanding of the relationship between compassion-related work lives and working environments will inform the development of targeted interventions aimed at enhancing working conditions, which, in turn, may improve the care experiences of care aides in LTC settings.

## Theoretical Framework

This study incorporates the compassion fatigue concept, and the job demand-resource model. The concept of compassion fatigue guides us to understand how healthcare workers’ mental health is affected by prolonged exposure to patients in distress. According to this concept, the inability to assist patients during critical stages of illness over an extended period of time increases the risk of compassion fatigue and burnout among caregivers (Figley, 2013). This concept argues that empathetic engagement, while essential for patient care, paradoxically heightens a caregiver’s vulnerability to compassion fatigue (Figley, 2013).

The job demand job resource model (JD-R) was introduced to understand burnout in the workplace and explain how the organizational environment influences employee’s well-being. According to this model, every job involves demand and resources. High job demands lead to physical and psychological exhaustion when they exceed an employee’s capacity to cope. Increasing job resources can reduce workload and prevent burnout by allowing employees to cope with demands and stay engaged with organizational goals (Bakker & Demerouti, 2018; Demerouti et al., 2001). For example, a study showed that the growing demand for care in hospitals and long working hours was associated with higher burnout levels (Xian et al., 2020). Additionally, low work resources, such as insufficient social support and lack of rewards, were also linked to increased burnout.

**Figure 1.**
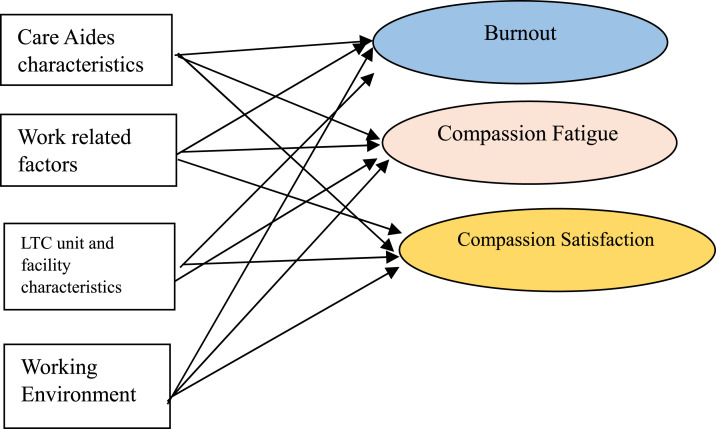
Conceptual framework for care aides’ burnout, compassion fatigue, and compassion satisfaction.

In this study, the job demands and resource model guided us to hypothesize how working environment factors, such as lack of staffing, increase job demands and exacerbate burnout while reducing compassion satisfaction (Figure 1).

## Methods

### Design and Data Sources

We conducted a cross-sectional analysis using data collected from the Translating Research in Elder Care (TREC) program during the COVID-19 pandemic (August 2021 to February 2022). TREC is a longitudinal research program that investigates the impact of the organizational environment on best practices and on the health of both staff and residents in residential LTC settings. This cross-sectional study followed the Strengthening the Reporting of Observational Studies in Epidemiology (STROBE) reporting guideline to ensure adequate reporting of study results (Von Elm et al., 2007).

### Study Sample

Our sample consisted of 760 care aides working in 98 care units within 28 LTC homes in Alberta, Canada. LTC homes were chosen using a stratified random sampling method, with strata being region, facility size, and the owner operator model. Care aides working in the selected LTC homes were invited in person to participate in the TREC care aide survey if they had a minimum of 3 months of experience working in the same care unit and completed at least six shifts every month. Trained interviewers completed virtual structured interviews with participants via Zoom. In our study, a total of 760 care aides (eligible sample *n* = 2241) voluntarily participated, yielding a response rate of 33.92%. This response rate in TREC was lower than usual, likely due to the survey being conducted during one of Alberta’s worst COVID years in 2021.

### Variables and Measures

The following variables and measures were collected using the care aide TREC survey. This survey identifies care aides’ compassion-related work life, work environment, and facility, unit, and individual characteristics (Table 1).

**Table 1.** Measures of Care Aides Professional Quality of Life, Working Environment, and Facility/Unit/Individual Characteristics.

Variables	Description	Items	Scoring	Cronbach’s alpha
A) Facility characteristics				
Zone	Zone where the facility is located		Edmonton	
			Calgary	N/A
Facility size	Number of beds in the facility	1	Small (<80 beds)	
			Medium (80–120 beds)	
			Large (>120 beds)	N/A
Owner operator model	Ownership model of the facility	1	Public not for profit	
			Voluntary not for profit	
			Private for profit	N/A
Number of COVID-19 outbreaks	Total number of COVID-19 outbreak in LTC homes	1	Continuous	N/A
B) Unit characteristics				
Type of unit	Name of the care aides working unit	1	General LTC	
			Others unit	N/A
Organizational citizenship behavior (OCB)	Mangers feeling a sense of responsibility to improve residents’ well-being	4	Yes	
			No	N/A
Total care hours per resident day	Total hours scheduled for registered nurses, licensed practical nurses, and care aides per resident day	1	Continuous	N/A
C) Demographics				
Age	Respondents age	1	<30 years	
			30–39 years	
			40–49 years	
			50–59 years	
			>60 years	N/A
Sex	Respondents sex identity	1	Male	
			Female	N/A
Marital status	Respondents marital condition	1	Single and never married, married and living together, widowed, separated, and divorced	N/A
English as first language	Respondents first language is English	1	Yes	
			No	N/A
Total family income	Total amount of money earned yearly by the family members	1	<$25000	
			$25000–$49999	
			$50 000–$74999	
			>$75000	N/A
D) Work-related factors				
Work short staffed	Opinion regarding staffing shortage	1	More or less every day Weekly	
			Monthly	
			Less than often	
			Never	N/A
Resident aggression	Aggressive or hostile behavior exhibited by residents in LTC	6	Sum of items on 2-point Likert scale (yes-no)	N/A
Working shift	Shift worked most of the time	1	Day	
			Evening	
			Night	N/A
Concurrent employment in jobs	Respondents working additional to current LTC job	1	Yes	
			No	N/A
E) Working environment (Alberta Context Tools)				
ACT Leadership	The actions of formal leaders in an organization (Unit) influence change and excellence in practice; items generally reflect emotionally intelligent leadership	6	Mean of items on 5-point Likert scale (Strongly disagree-agree)	0.78
ACT Culture	The way that “we do things” in our organization and unit; items generally reflect a supportive work culture	6	Mean of items on 5-point Likert scale (Strongly disagree-agree)	0.74
ACT Evaluation	The process of using data to assess group/team performances and to achieve outcomes in organizations or units	6	Mean of items on 5-point Likert scale (Strongly disagree-agree)	0.72
ACT Formal Interactions	Formal exchanges that occur between individuals working within an organization (Unit) through scheduled activities	4	5-point Likert frequency scale (Never-almost always). Recode 0 = no interaction, 1 = interaction. Take count of recoded items	0.42
ACT Informal Interactions	Information exchanges that occur between individuals within an organization that can promote the transfer of knowledge	9	5-point Likert frequency scale (Never-almost always). Recode 0 = no interaction, 1 = interaction. Take count of recoded items	0.69
ACT Social Capital	Active connections among people (bonding, bridging, and linking)	6	Mean of items on 5-point Likert scale (Strongly disagree-strongly agree)	0.71
ACT Structural Resources	The structural elements of an organization that facilitate the ability to assess and use knowledge	7	5-point Likert frequency scale (Never-almost always). Recode 0 = no resource, 1 = resource. Take count of recoded items	0.72
ACT Organizational Slack	The cushion of actual or potential resources which allows an organization to adapt successfully to internal pressures for adjustments or to external pressures for changes			
a) Staff	Enough staff to deliver quality of care	3	Mean of items on 5-point Likert scale (Strongly disagree-strongly agree)	0.91
b) Space	Adequate space to provide resident care	2	Mean of items on 5-point Likert scale (Strongly disagree-strongly agree)	0.78
c) Time	Time to do something extra for residents	4	Mean of items on 5-point Likert scale (Strongly disagree-strongly agree)	0.79
F) Professional Quality of Life (Compassion-related work life)				
Burnout (BO)	Repeated exposure of negative effects of working with traumatic patients develop gradually over the time	3	Sum of items on 5-point Likert scale (Never-very often)	0.75
Compassion fatigue (CF)	Negative effects of working with traumatic patients develop acutely and suddenly	3	Sum of items on 5-point Likert scale (Never-very often)	0.68
Compassion satisfaction (CS)	Positive effects of helping others or motivating them to engage in organizational goals	3	Sum of items on 5-point Likert scale (Never-very often)	0.73

#### Professional Quality of Life (ProQOL-9)

Care aide burnout, compassion fatigue, and compassion satisfaction were measured with the Professional Quality of Life-9 (ProQOL-9) (Galiana et al., 2020). ProQOL-9 is a 9-item validated instrument developed from the original and longer ProQOL IV and ProQOL V. Each of the three subscales is made up of three items on a 5-point Likert scale (1 = never to 5 = very often). Respondents were asked to identify how frequently they have had the experience described by each item in the past 30 days. Scores for each subscale are measured by summing up three items. The ProQOL-9 has been previously assessed for reliability and validity among palliative care professionals. Cronbach’s alpha scores were 0.83 for burnout, 0.82 for compassion fatigue, and 0.84 for compassion satisfaction (Galiana et al., 2020).

#### Work Environment (Organizational Context)

We used the Alberta Context Tool (ACT) to measure organizational context at the clinical microsystem (care unit) level (Estabrooks et al., 2009). The ACT has 10 concepts, including leadership, culture, evaluation (data feedback processes), formal and informal interactions, social capital, structural and electronic resources, and organizational slack (staffing, space, and time). Each item is measured on a 5-point Likert scale. Each scale score for leadership, culture, evaluation, social capital, and organizational slack in staffing, space, and time is obtained by taking the average of its items. Scores for formal interactions (range 0–4), informal interactions (range 0–9), and structural resources (range 0–7) are calculated by taking the count of recoded items. Previous studies have reported that the ACT is a valid and reliable instrument used for care aides in LTC with a Cronbach’s alpha being 0.70 for 8 of 10 concepts (Estabrooks, Morgan, et al., 2011; Estabrooks, Squires, et al., 2011).

### Statistical Analysis

We used descriptive analyses to examine the characteristics of care aides, units, and facilities. We used a two-level random intercept linear regression analysis (to adjust for the clustering of care aides within units) to examine the relationships between the work environment and care aides’ burnout, compassion fatigue, and compassion satisfaction. Our adjusted models controlled for care aide, unit, and facility characteristics (see Table 1). We initially developed a three-level model as our care aides nested in units and units nested in facilities (Hox et al., 2017). We excluded the facility level from our final model because it showed a low intra-class correlation at the facility level (ICC <0.001), thus leaving a two-level model. All 10 context variables were included in the models, as the variation inflation factors (VIF ≤ 1.8) indicated no issues of multi-collinearity (Harris, 2021). In our model, we used listwise deletion to deal with missing cases as there were less than 5% of missing data (Madley-Dowd et al., 2019; Schafer, 1999). We conducted linear regression analyses separately for each ProQOL subscale (burnout, compassion fatigue, and compassion satisfaction). The conditional pseudo-R^2^ value was used to assess model fitness, and the analyses were carried out with SPSS-28 version (IBM Corp N, 2017).

### Ethics

We obtained ethics approvals from the University of Alberta Health Research Ethics Board (Pro00037937).

## Results

Table 2 depicts care aide characteristics. Among the 760 participants, 91.0% of the care aides were female. The majority of participants (60%) were in the 40–59 years of age category; 73.3% of participants reported an annual total family income above $50,000. The majority of care aides (75.7%) did not speak English as their first language. Half of the participants (52.4%) worked on the day shift most of the time; 40.2% reported experiencing working short staffed on a weekly basis. Care aides, nurses, and licensed practical nurses dedicate an average of 2.7 hours per day for resident care. About 12% of care aides worked additional jobs outside of LTC homes. Table 3 shows the theoretical range, which represents the possible scores established by the measurement instruments, whereas the actual range represents the scores observed in this study. The theoretical range for ProQOL for each component was 3.0–15.0, although the actual ranges varied a little bit in this study. The average scores for compassion satisfaction, compassion fatigue, and burnout were 12.98 (±2.01), 5.52 (±2.25), and 6.95 (±2.72), respectively.

**Table 2.** Demographic Characteristics of Care Aides (*n* = 760).

Care aides characteristics	*n* (%)
Age	
<30 years	51 (6.7)
31–39 years	142 (18.7)
40–49 years	253 (33.3)
50–59 years	203 (26.7)
>60 years	111 (14.6)
Sex (*n* = 758)	
Male	68 (9.0)
Female	690 (91.0)
Marital status (*n* = 759)	
Single	135 (17.8)
Married and lived in together with another person as a couple but were not legally married	506 (66.7)
Divorced, separated, or widowed	118 (15.5)
English as first language (*n* = 759)	
Yes	188 (24.8)
No	571 (75.2)
Total family income (*n* = 731)	
< $25,000	15 (2.1)
$25,000–$49,999	180 (24.6)
$50,000–$74,999	267 (36.5)
> $75,000	269 (36.8)
Working in more than 1 LTC home	
Yes	84 (11.1)
No	676 (88.9)
Work short staffed (*n* = 756)	
More or less everyday	110 (14.6)
Weekly	304 (40.2)
Monthly	115 (15.2)
Less than often	179 (23.7)
Never	48 (6.3)
Working shift	
Day	398 (52.4)
Evening	303 (39.9)
Night	59 (7.8)
Resident aggression counts	2.95 (±1.72)
Facility characteristics	
Zone	
Edmonton	460 (60.5)
Calgary	300 (39.5)
Facility size	
Small (<80 beds)	78 (10.3)
Medium (80-120 beds)	126 (16.6)
Large (>120 beds)	556 (73.2)
Owner operator model	
Public	144 (18.9)
Private	222 (29.2)
Voluntary	394 (51.8)
Unit characteristics	
Type of unit (*n* = 756)	
General LTC	338 (44.7)
Others unit	418 (55.3)
Total care hours per resident day	2.87 (±1.17)

**Table 3.** Care Aides’ Responses on Working Environment and Professional Quality of Life.

	Mean (SD)	Actual ranges	Theoretical ranges
Working environment (ACT)			
Leadership	4.03 (0.52)	1.0–5.0	1.0–5.0
Culture	4.12 (0.47)	2.0–5.0	1.0–5.0
Evaluation	3.83 (0.54)	1.0–5.0	1.0–5.0
Formal Interactions	1.50 (0.84)	00–4.0	00–4.0
Informal Interactions	4.23 (1.69)	00–9.0	00–9.0
Social Capital	4.02 (0.50)	2.0–5.0	1.0–5.0
Structural Resources	2.92 (1.61)	00–7.0	00–11.0
Organizational Slack-Staffing	3.03 (1.10)	1.0–5.0	1.0–5.0
Organizational Slack-Space	3.69 (1.09)	1.0–5.0	1.0–5.0
Organizational Slack-Time	3.56 (0.82)	1.0–5.0	1.0–5.0
Professional Quality of Life (ProQOL-9)			
Compassion fatigue (CF)	5.52 (2.25)	3.0–14.0	3.0–15.0
Burnout (BO)	6.95 (2.72)	3.0–15.0	3.0–15.0
Compassion satisfaction (CS)	12.98 (2.01)	4.0–15.0	3.0–15.0

Table 4 presents the results of the two-level multilevel regression. Burnout was negatively associated with culture (B = −0.907, *p* = <0.001, 95% CI = −1.422 to −0.391), organizational slack in staffing (B = −0.470, *p* = <0.001, 95% CI = −0.676 to −0.263), and organizational slack in time (B = −0.338, *p* = .019, 95% CI = −0.621 to −0.055). Compassion fatigue was negatively associated with structural resources (B = −0.148, *p* = .019, 95% CI = −0.272 to −0.023) and organizational slack in staffing (B = −0.248, *p* = .007, 95% CI = −0.431 to −0.066) but positively associated with formal interactions (B = 0.246, *p* = .025, 95% CI = 0.029 to 0.463). Compassion satisfaction was positively associated with culture (B = 0.998, *p* = < 0.001, 95% CI = 0.586 to 1.409). Table 4 also reveals that care aides working evening shifts were significantly more likely to experience burnout (B = 0.717, *p* = .043, 95% CI = 0.022 to 1.412) compared to night shift workers. Resident aggression was positively associated with care aide burnout (B = 0.350, *p* = <.001, 95% CI = 0.241 to 0.460) and compassion fatigue (B = 0.359, *p* = <.001, 95% CI = 0.262 to 0.455) but negatively associated with compassion satisfaction (B = −0.137, *p* = .002, 95% CI = −0.439 to −0.230).

**Table 4.** Compassion-Related Work Life Among Care Aides in LTC Homes.

Random effect	Burnout		Compassion fatigue		Compassion satisfaction	
	B(SE)	95% CI	B(SE)	95% CI	B(SE)	95% CI
Unit (null model)	6.979 (.122)	6.233 to 7.222	5.532 (.095)	5.342 to 5.722	12.981 (.089)	12.804 to 13.159
ICC unit (null model)	.451 (.213)	.179 to 1.138	.205 (.126)	.062 to .683	.229 (.106)	.093 to .566
Unit (full model)	12.584 (1.504)	9.623 to 15.545	5.461 (1.285)	2.931 to 7.799	7.500 (1.196)	5.146 to 9.854
ICC unit (full model)	.144 (.139)	.017 to .995	.004 (.093)	1.470 to 9.285	.078 (.090)	.008 to .751

Fixed effects		Burnout (conditional pseudo-*R*^2^ = 0.305)			Compassion fatigue (conditional pseudo-*R*^2^ = 0.191)			Compassion satisfaction (conditional pseudo-*R*^2^ = 0.199)		
		B(SE)	95% CI	*p*	B(SE)	95% CI	*p*	B(SE)	95% CI	*p*
Age	Continuous	−.059 (.047)	−.151 to .032	.206	.058 (.041)	−.023 to .138	.159	.072 (.037)	−.001 to .145	.053
Sex	Male	−.127 (.321)	−.757 to .503	.693	−.254 (.284)	−.811 to .303	.372	−.449 (.256)	−.953 to .054	.080
	Reference category: Female									
English as first language	Yes	.426 (.239)	−.044 to .896	.076	.019 (.211)	−.396 to .434	.929	.244 (.191)	−.132 to .619	.203
	Reference category: No									
Marital status	Married and live together	.090 (.258)	−.416 to .597	.726	.537 (.228)	.089 to .986	.**019**	.055 (.206)	−.349 to .460	.800
	Divorced, widowed, and separated	−.398 (.337)	−1.061 to .264	.238	.393 (.298)	−.192 to .979	.188	.019 (.270)	−.510 to .549	.942
	Reference category: Single									
Zone	Edmonton	−.185 (.228)	−.640 to .269	.419	.007 (.187)	−.365 to .379	.970	−.022 (.181)	−.381 to .337	.903
	Reference category: Calgary									

Fixed effects		Burnout			Compassion fatigue			Compassion satisfaction		
		B(SE)	95% CI	*p*	B(SE)	95% CI	*p*	B(SE)	95% CI	*p*
Working in more than 1 LTC home	Yes	.182 (.298)	−.404 to .768	.542	−.096 (.264)	−.613 to .422	.725	−.062 (.238)	−.530 to .406	.796
	Reference category: No									
Shift work	Day	.659 (.353)	−.034 to 1.352	.064	.499 (.313)	−.116 to 1.113	.111	−.054 (.282)	−.608 to .500	.847
	Evening	.717 (.354)	.022 to 1.412	**.043**	.420 (.314)	−.197 to 1.037	.186	−.084 (.283)	−.640 to .472	.767
	Reference category: Night									
Annual income	< $25000	−1.309 (.656)	−2.597 to −.022	.046	.112 (.579)	−1.025 to 1.249	.847	−.225 (.524)	−1.245 to .804	.668
	$25000 to $49 999	−.237 (.255)	−.736 to .267	.358	.054 (.226)	−.390 to .498	.812	.029 (.204)	−.372 to .429	.889
	$50 000 to 74 999	−.382 (.219)	−.811 to .051	.084	−.471 (.193)	−.849 to −.089	**.016**	.266 (.175)	−.077 to .610	.129
	Reference category: > $75 000									
Owner operator model	Public	−.043 (.313)	−.667 to .581	.891	.048 (.254)	−.459 to .554	.852	−.077 (.247)	−.416 to .570	.755
	Private for profit	−.179 (.247)	−.685 to .314	.461	.317 (.200)	−.085 to .719	.120	−.115 (.197)	−.509 to .279	.561
	Reference category: Voluntary									
Facility size	Small	.401 (.348)	−.290 to 1.092	.252	.797 (.286)	.229 to 1.365	**.008**	−.053 (.275)	−.494 to .600	.848
	Medium	.174 (.359)	−.556 to .897	.640	.252 (.287)	−.329 to .834	.367	−.176 (.285)	−.750 to .397	.539
	Reference category: Large									
Unit type	General LTC	−.098 (.218)	−.533 to .338	.656	−.051 (.175)	−.404 to .301	.736	.064 (.172)	−.280 to .408	.711
	Reference category: Others									
Total care hours per resident day	Continuous	.009 (.092)	−.175 to .194	.919	−.048 (.073)	−.196 to .099	.514	−.094 (.072)	−.239 to .051	.200
Organizational citizenship behavior (OCB)	Continuous	.232 (.196)	−.158 to .621	.240	−.047 (.160)	−.365 to .271	.768	−.251 (.155)	−.559 to .057	.108
COVID-19 outbreaks	Continuous	−.035 (.057)	−.149 to .078	.534	.102 (.045)	.011 to .193	.**029**	.051 (.045)	−.038 to .140	.257
Resident Aggression	Continuous	.350 (.056)	.241 to .460	**<.001**	.359 (.049)	.262 to .455	**<.001**	−.137 (.045)	−.439 to −.230	.**002**
Working environment (Alberta Context Tools)										
	Leadership	−.241 (.213)	−.659 to .178	.260	.005 (.189)	−.366 to .375	.996	.105 (.170)	−.234 to .435	.539
	Culture	−.907 (.262)	−1.422 to −.391	**<.001**	−.453 (.231)	−.907 to .002	.055	.998 (.209)	.586 to 1.409	**<.001**
	Evaluation	.119 (.208)	−.290 to .527	.564	.122 (.184)	−.240 to .483	.518	−.011 (.166)	−.337 to .316	.949
	Formal interactions	.162 (.125)	−.084 to .407	.197	.246 (.110)	.029 to .463	**.025**	−.138 (.100)	−.334 to .058	.168
	Informal interactions	.108 (.063)	−.016 to .233	.088	.104 (.056)	−.006 to .214	.067	.004 (.051)	−.095 to .104	.934
	Social capital	−.278 (.214)	−.698 to .141	.193	−.188 (.188)	−.558 to .182	.319	.283 (.171)	−.052 to .618	.097
	Structural resources	−.117 (.072)	−.259 to .024	.103	−.148 (.063)	−.272 to −.023	.**019**	.087 (.057)	−.026 to .199	.131
	Organizational slack in staffing	−.470 (.105)	−.676 to −.263	**<.001**	−.248 (.093)	−.431 to −.066	**.007**	.128 (.084)	−.037 to .293	.128
	Organizational slack in space	.011 (.091)	−.168 to .189	.903	.052 (.080)	−.106 to .209	.256	.044 (.073)	−.099 to .186	.549
	Organizational slack in time	−.338 (.144)	−.621 to −.055	**.019**	−.000 (.128)	−.251 to .220	.978	.099 (.115)	−.127 to .325	.392

*Note.* ICC = intra-class correlation, 95% CI = 95% confidence interval, *R*^2^ = proportion of variance explained by both the fixed and random factors.

## Discussion

Our study fills a critical gap in the literature by providing insights into which elements of the working environment influence compassion fatigue among workers in LTC settings. We found that a supportive work culture was strongly associated with higher compassion satisfaction and lower burnout among care aides. In contrast, compassion fatigue was linked to inadequate structural and staffing resources. Furthermore, care aides who perceived staffing shortages or insufficient time to complete tasks reported significantly higher levels of burnout, highlighting the impact of resource constraints on their psychological well-being.

A working environment can be called supportive or positive when it promotes a culture of teamwork, recognition, autonomy, and professional development (Estabrooks et al., 2009). In our study, care aides reported burnout and compassion fatigue may be due to a lack of supportive organizational cultures in their workplace. When employees feel unsupported, particularly in emotionally demanding roles like caregiving, it can contribute to feelings of isolation and disempowerment, which are key factors in burnout (Maslach & Leiter, 2016). A systematic review of organizational culture among nurses showed that a positive or supportive organizational culture is significantly associated with reducing work-related stress (Kiptulon et al., 2024). Similarly, critical care nurses reported that workplace recognition significantly enhanced compassion satisfaction and decreased burnout (Kelly & Lefton, 2017). While our study did not reveal particular organizational culture elements associated with burnout and compassion fatigue, future research should concentrate on the components of supportive cultures that address these challenges. Furthermore, interventions such as team-building, recognition programs, and professional development opportunities should be designed and tested for their usefulness in improving care aides’ well-being and job satisfaction.

Adequate staffing is an important component of a positive work environment and has long been identified as a major contributor to employee burnout. A substantial body of research suggests that insufficient staffing levels place an extra burden on current employees, leading to emotional exhaustion and subsequent burnout (Chemali et al., 2022; Konstantinou et al., 2018; Kowalski et al., 2010; Søvold et al., 2021; Toh et al., 2012; Tsolekile et al., 2015). The job demands–resources model of burnout argues that when job demands are high and resources (staff) are low, stress and burnout increase (Demerouti et al., 2001). Staffing in Canadian long-term care homes is particularly difficult, with a heavy reliance on part-time caregivers. A study recommends increasing permanent staff to address staffing shortages in long-term care facilities (Franzosa et al., 2022).

We argue that care aides may feel compassion fatigue due to their inability to help residents because of increased workload and insufficient staff. This was supported by another finding from our study, where care aides’ perception of a lack of time to implement best practices was associated with higher levels of burnout. Having perception of sufficient time for extra tasks and collaborative discussions about best practices were linked to lower levels of burnout (Cooper et al., 2016) and increased job satisfaction (Chamberlain et al., 2016). Heavy workloads limit care aides’ opportunities for meaningful interactions with residents (Mallidou et al., 2013). Because of their substantial workloads, care aides tend to complete their duties without engaging in meaningful interactions with residents (Wiersma, 2012).

Our study indicates that a scarcity of structural resources, including clinical guidelines, textbooks, and notice boards may contribute to increased levels of compassion fatigue. While compassion-related research is limited in LTC working environments, some studies found correlations between structural resources and burnout in other settings. For example, lack of structural resources in LTC homes was correlated with burnout among care aides (Chamberlain et al., 2017). During the COVID-19 pandemic, clinical guidelines served as psychological safeguards for nurses (Maiorano et al., 2020), whereas emergency healthcare workers reported elevated levels of burnout when faced with insufficient resources (Blanchard et al., 2022).

We reported that care aides who participated in formal interactions, such as team meetings and family conferences, experienced higher levels of compassion fatigue. While no direct evidence explicitly linking formal interactions to this outcome has been identified in previous research, studies highlighted that excessive empathetic engagement with patients and their families contributed to significant emotional exhaustion (Anderson et al., 2019) and compassion fatigue among healthcare workers (Nishihara et al., 2022). Engaging in conversations with family members about a patient’s declining health, persistent pain, or approaching end-of-life can be emotionally challenging for healthcare workers, often evoking feelings of helplessness, profound grief, and emotional exhaustion. Further research is needed to explore the direct impact of these formal interactions on caregiver well-being.

## Limitations

Our study had some important limitations. First, generalizations can only be made for care aides working in LTC homes, similar to those reported here. Second, we may have had a significant response bias because of low response rate. Third, as the study is cross-sectional, we were unable to make any causal claims regarding the relationships between the LTC working environment and compassion fatigue, burnout, and compassion satisfaction. Fourth, survey data may be subject to recall biases or social desirability biases. The study is further limited to the context of the COVID-19 pandemic, which may have unique effects on the working environment and psychological outcomes of care aides.

## Conclusion

Less supportive work culture was correlated with higher burnout and lower compassion satisfaction, while insufficient structural or staffing resources were linked to increased compassion fatigue. Perceptions of insufficient staffing and time constraints were also associated with higher levels of burnout. These findings highlight the critical importance of a positive and supportive work environment in preventing compassion fatigue and burnout in this frontline caregiving workforce through future intervention. A more stable and resilient workforce is likely to provide higher quality care to residents and enhance workforce retention.
